# Tracking Public Attitudes Toward COVID-19 Vaccination on Tweets in Canada: Using Aspect-Based Sentiment Analysis

**DOI:** 10.2196/35016

**Published:** 2022-03-29

**Authors:** Hyeju Jang, Emily Rempel, Ian Roe, Prince Adu, Giuseppe Carenini, Naveed Zafar Janjua

**Affiliations:** 1 Department of Computer Science University of British Columbia Vancouver, BC Canada; 2 British Columbia Centre for Disease Control Vancouver, BC Canada; 3 School of Population and Public Health University of British Columbia Vancouver, BC Canada; 4 Centre for Health Evaluation and Outcome Sciences St Paul's Hospital Vancouver, BC Canada

**Keywords:** COVID-19, vaccination, Twitter, aspect-based sentiment analysis, Canada, social media, pandemic, content analysis, vaccine rollout, sentiment analysis, public sentiment, public health, health promotion, vaccination promotion

## Abstract

**Background:**

The development and approval of COVID-19 vaccines have generated optimism for the end of the COVID-19 pandemic and a return to normalcy. However, vaccine hesitancy, often fueled by misinformation, poses a major barrier to achieving herd immunity.

**Objective:**

We aim to investigate Twitter users’ attitudes toward COVID-19 vaccination in Canada after vaccine rollout.

**Methods:**

We applied a weakly supervised aspect-based sentiment analysis (ABSA) technique, which involves the human-in-the-loop system, on COVID-19 vaccination–related tweets in Canada. Automatically generated aspect and opinion terms were manually corrected by public health experts to ensure the accuracy of the terms and make them more domain-specific. Then, based on these manually corrected terms, the system inferred sentiments toward the aspects. We observed sentiments toward key aspects related to COVID-19 vaccination, and investigated how sentiments toward “vaccination” changed over time. In addition, we analyzed the most retweeted or liked tweets by observing most frequent nouns and sentiments toward key aspects.

**Results:**

After applying the ABSA system, we obtained 170 aspect terms (eg, “immunity” and “pfizer”) and 6775 opinion terms (eg, “trustworthy” for the positive sentiment and “jeopardize” for the negative sentiment). While manually verifying or editing these terms, our public health experts selected 20 key aspects related to COVID-19 vaccination for analysis. The sentiment analysis results for the 20 key aspects revealed negative sentiments related to “vaccine distribution,” “side effects,” “allergy,” “reactions,” and “anti-vaxxer,” and positive sentiments related to “vaccine campaign,” “vaccine candidates,” and “immune response.” These results indicate that the Twitter users express concerns about the safety of vaccines but still consider vaccines as the option to end the pandemic. In addition, compared to the sentiment of the remaining tweets, the most retweeted or liked tweets showed more positive sentiment overall toward key aspects (*P*<.001), especially vaccines (*P*<.001) and vaccination (*P*=.009). Further investigation of the most retweeted or liked tweets revealed two opposing trends in Twitter users who showed negative sentiments toward vaccines: the “anti-vaxxer” population that used negative sentiments as a means to discourage vaccination and the “Covid Zero” population that used negative sentiments to encourage vaccinations while critiquing the public health response.

**Conclusions:**

Our study examined public sentiments toward COVID-19 vaccination on tweets over an extended period in Canada. Our findings could inform public health agencies to design and implement interventions to promote vaccination.

## Introduction

The development and approval of COVID-19 vaccines have generated optimism for the end of the COVID-19 pandemic and a return to normalcy. Current data from clinical trials and real-world studies show that these vaccines have high efficacy and effectiveness in preventing infection and severe disease [1-3]. Since the start of vaccination in Canada and many countries with high vaccine coverage, deaths among vaccinated groups have plummeted, and most new infections and hospitalizations are occurring among unvaccinated individuals, although recently more breakthrough infections have been reported with the Omicron variant. However, the overall impact on reduction in transmission, morbidity, mortality, as well as the easing of restrictions will depend on vaccine coverage and uptake. Vaccine acceptance and uptake remains one of the most important public health concerns in many countries. Vaccine hesitancy—often fueled by misinformation surrounding the importance, safety, or effectiveness of the vaccine—poses a major barrier to achieving herd immunity [4]. The failure to eradicate other infectious diseases including polio, for instance, has been blamed on conspiracy theories that fueled vaccine hesitancy [5]. The same concern is now threatening the successful implementation of mass COVID-19 vaccination campaigns. A systematic review of 126 surveys published before November 2020 showed an increasing worldwide hesitancy toward the COVID-19 vaccine [6].

In an era of misinformation and disinformation, identifying and understanding vaccine hesitancy are key to containing the spread of the virus and crucial to framing public health messaging [4]. In addition to individual views on the vaccine, negative discourse and misinformation related to it on social media could create doubt. Similar to infection, misinformation on social media spreads within the social network (echo chambers) and could affect acceptance among people. A recent study noted a decline in vaccine acceptance from exposure to misinformation on social media [7]. In addition to broader vaccine hesitancy, vaccine hesitancy in local areas or networks could also increase the risk of transmission within the community even if the broader population is immune. Tackling misinformation about the vaccine on social media will be critical to achieving herd immunity and a path out of the pandemic.

Various studies have investigated people’s attitudes toward COVID-19 vaccination and addressed vaccine misinformation in social media. Puri et al [8] discussed digital health strategies to handle vaccine misinformation on social media such as framing messages and leveraging celebrities. Chou and Budenz [9] reported that people’s reactions to the COVID-19 vaccines are emotionally charged, and emphasized the role of emotion in vaccine communication efforts to address vaccine hesitancy. Thelwall et al [10] manually categorized 446 COVID-19 vaccine–hesitant tweets in English (March 10 to December 5, 2020) into 14 classes on the basis of what types of vaccine hesitancy information are shared. Some of these classes were efficacy, safety, and conspiracy. Hussain et al [11] performed sentiment analysis on Facebook and Twitter data to understand public sentiments toward COVID-19 vaccines in the United Kingdom and the United States. Guntuku et al [12] reported geographical and temporal variation in concerns about COVID-19 vaccines in the United States by using topic modeling. Kwok et al [13] analyzed tweets from Australian users by using topic modeling and sentiment analysis. Griffith et al [14] conducted content analysis on tweets from Canada, posted between December 10 and December 23, 2020, and identified reasons underlying vaccine hesitancy among Twitter users in Canada. Although prior research investigated public attitudes toward COVID-19 vaccination, to our knowledge, there has not been much research examining public sentiments toward COVID-19 vaccination over an extended period following the start of vaccine rollout. In addition, no prior work has investigated the most influential tweets.

In this paper, we investigate Twitter users’ attitudes toward COVID-19 vaccination in Canada from December 2020 to May 2021. More specifically, we examined sentiments toward certain aspects related to COVID-19 vaccination, which were chosen by our public health domain experts, and show how those sentiments change over time. In addition, we observe what the most retweeted or liked tweets discuss and how their sentiments are. For these analyses, we use weakly supervised aspect-based sentiment analysis (ABSA) [15], a natural language processing technique that allows identifying sentiments for aspects. Our findings could inform the design and implementation of public health interventions to promote vaccination, toward the goal of ending the pandemic.

## Methods

### Methods Overview

**Figure 1:**
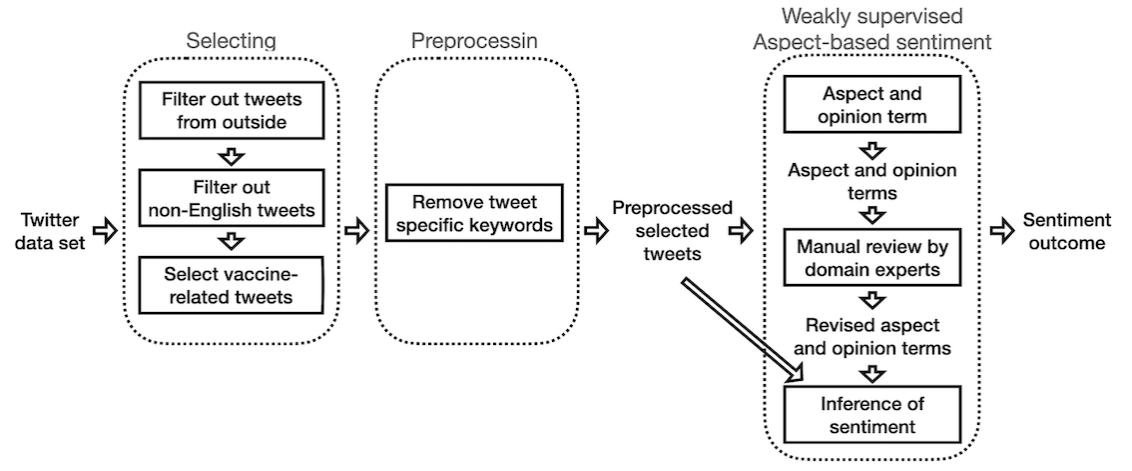
Study overview.

The overview of our process is displayed in Figure 1. We first selected COVID-19 vaccination–related tweets and then preprocessed them. Then, based on these tweets, we performed weakly supervised ABSA.

### Data and Preprocessing

We used a public Twitter data set about the COVID-19 pandemic, which was curated by Chen et al [16], using numerous COVID-19–related case-insensitive keywords such as “coronavirus,” “COVID-19,” and “pandemic.” Data collection started on January 28, 2020 (tweets from January 21, 2020), and is still ongoing. For the analysis in this paper, we collected tweets from December 2020 to May 2021, which spans from the beginning of vaccine rollout in Canada to the present.

From the data set, we extracted tweets originating in Canada and written in English by using tweet metadata and the spacy-langdetect toolkit (version 0.1.2) [17]. In addition, to focus on COVID-19 vaccination, we selected vaccine-related tweets using keywords (eg, “vaccine,” “vaccination,” “immunity,” “immune,” “rollout,” “mrna,” and “side effect”) defined by our domain experts. Our experts selected these keywords on the basis of public questions they received at the British Columbia Centre for Disease Control, which is responsible for vaccine programs and vaccine promotion. The original COVID-19 data set collected by Chen et al [16] contains 678,456,379 tweets for December 2020 to May 2021. Among them, the number of English tweets in Canada was 193,071. Of these, the number of the tweets that contain our search keywords was 21,821. As a result, 21,821 tweets were used for analysis. Among these, 10,202 tweets were original tweets, 6664 tweets were retweets or quoted tweets, 4955 tweets were replies to other tweets, and 112 tweets were both replies and quoted tweets. Of note, a quoted tweet is a type of retweet that contains an additional comment of the user. To remove tweet-specific keywords and URLs, we used the tweet-preprocessor toolkit (version 0.5.0) [18]. We did not remove hashtags and mentions because they are often part of a sentence, and could be informative for our work.

### ABSA

We used ABSApp [19], a weakly supervised ABSA human-in-the-loop system, which enables public health domain experts to modify aspect and opinion terms that were automatically extracted from the corpus by the system. More specifically, ABSApp automatically extracts both aspect and opinion terms from the data set without any manual labeling as the initial step and then allows humans to revise these terms. Then, based on these terms, the system automatically infers sentiment appearing in the data toward the aspect terms (Figure 1). In comparison to this weakly supervised ABSA system, a supervised method uses human labeled data and an unsupervised method does not use human efforts at all.

The aspect terms represent aspects we want to compute sentiment for, such as “pfizer” or “side effects.” The opinion terms are those that can be used for inference of sentiment; for example, “sore” for a negative sentiment and “immune” for a positive sentiment. The aspect and opinion terms can be domain-specific; hence, it is important to allow domain experts to edit them rather than just using automatically extracted general terms. These modified aspect and opinion terms were used for inference of sentiments for aspects.

To extract aspect and opinion terms in an unsupervised way using the ABSApp system first, we used 9239 tweets from December 2020 to February 2021. We used only the subset of the data for computational efficiency. The original study by Pereg et al [19] extracted terms from 75% of 2 data sets that consist of 5841 and 3614 sentences, respectively, which were much smaller than our data used for term extraction. Previous work [20] showed experimental results suggesting that increasing amounts of data beyond a certain point are unlikely to significantly increase the number of aspect terms when using ABSApp. In addition, opinion terms are not expected to differ much between February 2021 and May 2021 as they are the terms that indicate the sentiment of the tweets. The system automatically extracted 108 aspect terms and 6793 opinion terms.

Automatically extracted terms were manually reviewed and edited by 2 public health experts. Each expert reviewed and edited the whole list of aspect terms and opinion terms separately. The discrepancies in edits were addressed differently for the aspect terms and the opinion terms. For the aspect terms, we did not discard differences and selected all terms including discrepancies because an aspect term could be meaningful if at least one expert found it useful. For the opinion terms, we excluded the terms that did not agree because those opinion terms would not be a strong indicator of a sentiment if the experts did not agree. This manual edition process resulted in 170 aspect terms and 6775 opinion terms, and they were used for the sentiment inference step. Among the aspect terms, our experts selected 20 key aspects more relevant to COVID-19 vaccination, for which we report results in this paper.

In addition, we investigated most liked or retweeted tweets to determine what they discuss and which sentiment they convey. We first identified original tweets that were not retweets, quoted tweets, or replies to other tweets. Tweet metadata such as *retweeted_status*, *is_quote_status*, and *in_reply_to_status_id* were used. Then, we sorted the original tweets by their *retweet_count* and *favorite_count* (Twitter changed the “favorite” button to a “like” button; hence, hereinafter, we have used these two terms interchangeably). To observe the discourse in the top-ranked original tweets, we computed the frequency of nouns appearing in the tweets. For this computation, we first tokenized preprocessed tweets that did not contain tweet-specific keywords, URLs, and hashtags, and tagged part-of-speech. For both tokenization and part-of-speech tagging, the spacy toolkit (version 2.1.8) [21] was used. Scripts for preprocessing and analysis are available on the internet [22].

## Results

**Figure 2:**
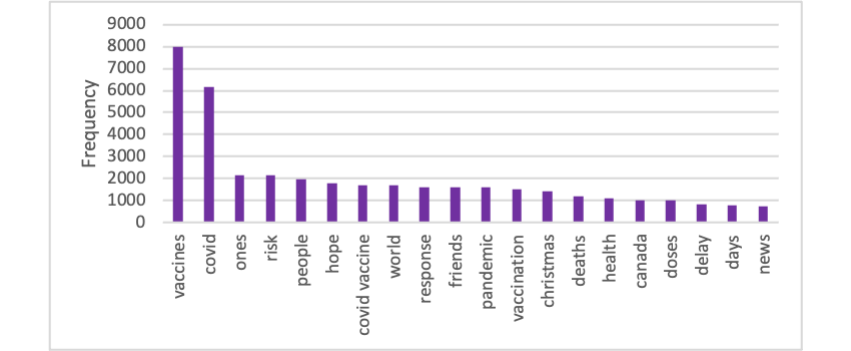
Most frequently appearing aspects in the data, extracted automatically by the system.

Figure 2 shows the most frequently appearing aspect terms that were automatically extracted by the system. “vaccines” and “covid” were the most common aspects, probably because they were used for filtering out non–vaccine-related tweets during the data collection. The other words describe the discussion in this data set. People discussed aspects such as “hope,” “people,” “friends,” “world,” “deaths,” and “delay” together with COVID-19 vaccines. The less meaningful aspects (eg, “ones”) are included as these aspects were automatically extracted without any manual intervention.

**Figure 3:**
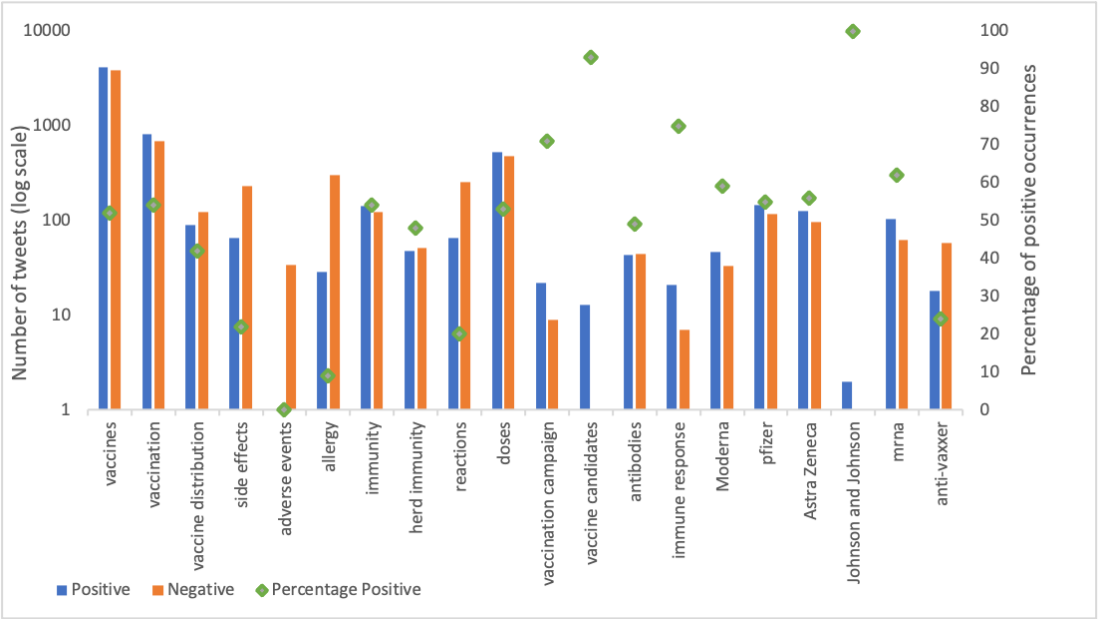
Results of aspect-based sentiment analysis. x-axis: selected aspects, y-axis (left): number of positive occurrences and number of negative occurrences in log scale; secondary y-axis (right): percentage of positive occurrences.

The sentiment outcomes of the aspects most relevant to COVID-19 vaccines, which were selected by public health experts, are shown in Figure 3. Overall, the sentiment toward “vaccines,” “vaccination,” and specific vaccine brands such as “pfizer” and “moderna” show similar levels of positive and negative sentiments. A more negative sentiment was observed with “vaccine distribution,” “side effects,” “allergy,” “reactions,” and “anti-vaxxer.” A more positive sentiment was observed toward “vaccination campaign,” “vaccine candidates,” and “immune response.”

**Figure 4:**
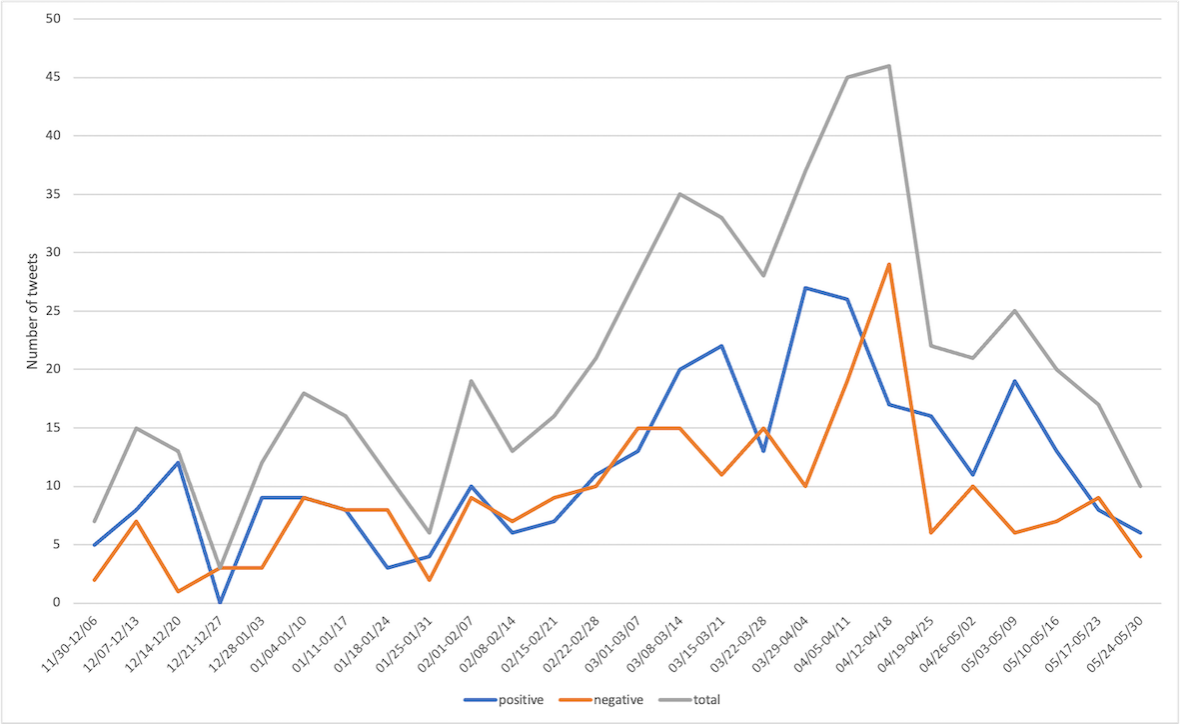
Sentiment changes over time for COVID-19 vaccination. Primary y-axis (left): number of total occurrences; secondary y-axis (right): percentage of positive occurrences.

Figure 4 shows the change in sentiment over time toward “vaccination.” First, we observed that the discussion about vaccination increased drastically in March and peaked in mid-April. Thereafter, it decreased.

Tables 1 and 2 show the distribution of how many times the original tweets were retweeted and liked by other users, respectively. As expected, large number of tweets were not retweeted or liked, while a small number of tweets had a large number of retweets and likes.

**Figure 5:**
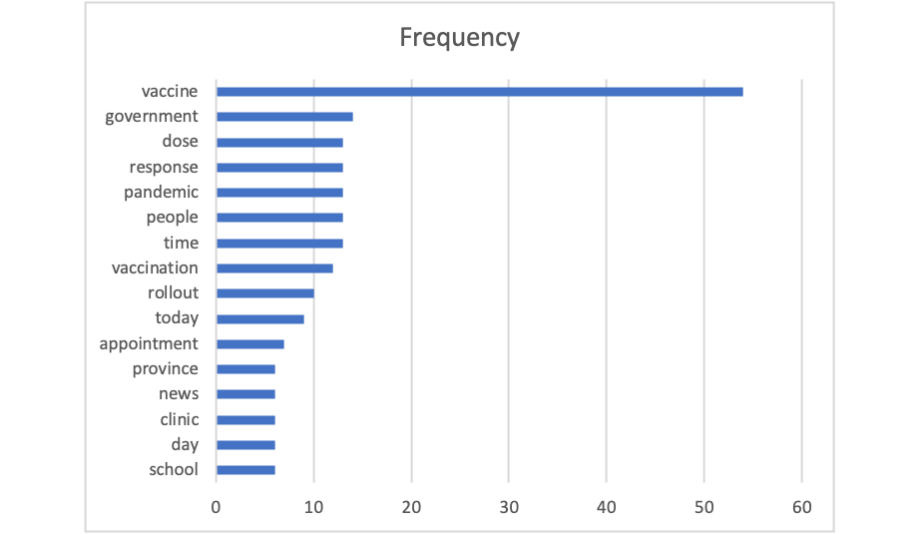
Terms that occur in more than 5 documents among the 100 most retweeted original tweets.

Figure 5 displays nouns that appeared in more than 5 tweets among the 100 most retweeted original tweets. These tweets were the same as the 100 most liked original tweets. In addition, Table 3 lists top 3 most liked and retweeted tweets. These data show that the most influential tweets were related to vaccine rollout, distribution, or administration and people’s hope and wish to end the pandemic situation.

**Figure 6:**
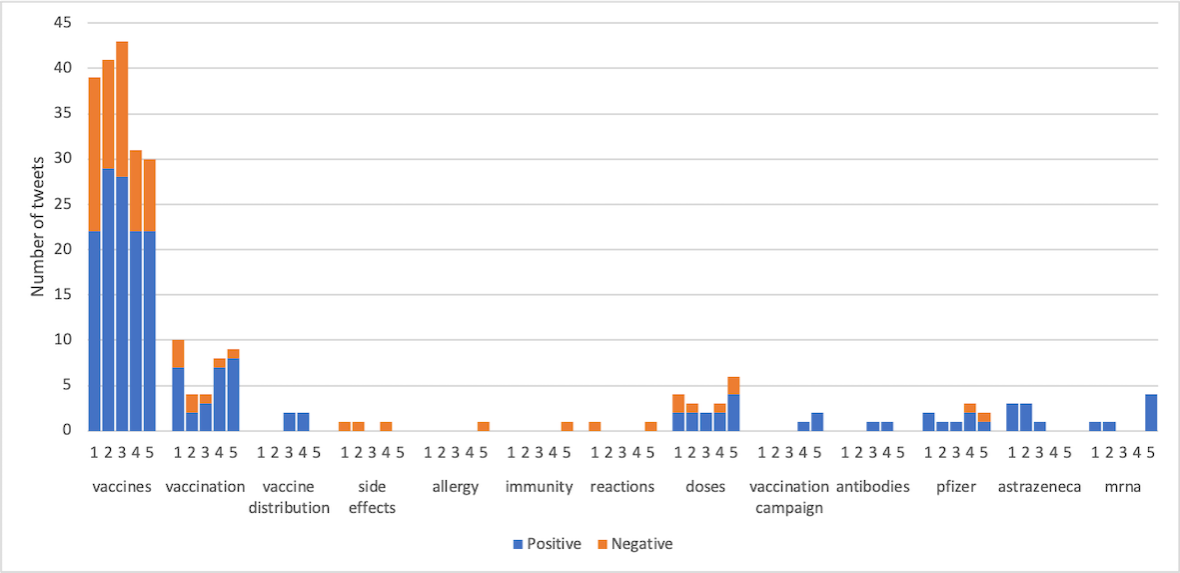
Sentiment toward certain aspects observed in the 500 most influential (most liked) tweets (1: top 100 tweets, 2: 101st-200th, 3: 201st-300th, 4: 301st-400th, 5: 401st-500th, y-axis: number of tweets).

Figure 6 shows the sentiment of 500 most liked tweets toward the key aspects selected by our public health experts. Compared to the sentiment of all the remaining tweets, these tweets show a significantly more positive sentiment overall about the key aspects (*P*<.001), especially vaccines (*P*<.001) and vaccination (*P*=.009), as revealed through chi-square analysis.

**Table 1:** Frequency distribution of original tweets being retweeted.

Number of times a tweet is retweeted	Frequency (number of tweets)
0	7033
1-50	3040
51-100	50
101-200	46
201-2169	33

**Table 2:** Frequency distribution of original tweets being liked.

Number of times a tweet is liked	Frequency (number of tweets)
0	4804
1-50	4863
51-100	238
101-200	126
201-400	79
401-800	52
801-7304	40

**Table 3:** Top 3 most liked original tweets (identifying information was removed).

Tweets	Likes, n	Retweets, n	Followers of the author, n
Do you know why they haven’t lifted the pandemic “emergency” yet? The first sentence on this pfizer website has an answer: none of the vaccines have been approved. They are only authorized for use in an “emergency”. No emergency, no experimental vaccines.	4306	2169	256,484
We have a hyperinfectious Covid variant circulating in the community, a vaccine shortage and are just on the verge of restoring contact tracing. If we pursue a #COVIDzero strategy we can be out of this by March. If we don’t we can forfeit our sacrifices & see a huge 3rd wave.	4169	1329	20,352
Vaccines sitting in freezers in Ontario: 985,132 Total vaccines administered today: 59,567 please stop playing games with our lives. Implement #PaidSickDays, ramp up vaccine administrations, and a real lockdown. Mockdown 3.0 isn’t cutting it.	2926	1061	23,923

## Discussion

### Principal Findings

In this study, we examined sentiments expressed toward COVID-19 vaccination on Twitter in Canada from December 2020 to May 2021. Major aspects included risk, hope, deaths, and delay. Positive sentiments were expressed for “vaccination campaign,” “vaccine candidates,” and “immune response,” while negative sentiments were expressed for issues related vaccine distribution, adverse effects (safety), and antivaccination. As expected, there was a small number of tweets that were retweeted multiple times. Within this group, we found antivaccination tweets, which were retweeted multiple times to a large number of people. Tackling misinformation spread by this small number of posts could have a large impact on countering vaccine misinformation.

The aspect “risk” in our most frequent aspects (Figure 2) suggests that people who were concerned about the risk of COVID-19 considered vaccination a solution. There were also tweets about the risk of side effects of vaccination, but mostly the conversations were centered around the risk of COVID-19 outweighing the risk of vaccination. This is aligned with prior work, which revealed that the safety category was ranked in the top 3 among 446 COVID-19 vaccine–hesitant tweets between March 10 and December 5, 2020 [10]. Survey data on vaccine acceptance also indicate an association between concerns about vaccine safety and a low intention to receive a COVID-19 vaccine [23-25]. A similar finding was observed in the study by Lyu et al [26], which showed that “trust” was the most dominant emotion in tweets from March 11, 2020, to January 31, 2021. This highlights the need to address safety concerns about vaccines to increase vaccine uptake. Other frequently discussed aspects “delay” and “hope” in our data indicate that people were worried about the delay in vaccine rollout in Canada but still considered vaccines as the option to end the pandemic. These aspects highlight frustration from twitter users in the delayed vaccine supply in Canada compared to those in the United States and in other countries.

Our results show that the largest segment of negative sentiment was toward vaccine adverse effects including “allergy,” “side effects,” and “reactions” (Figure 3). This finding is similar to prior work that showed that the largest public concern about COVID-19 vaccines was safety. Eibensteiner et al [27] reported that 41.7% of Twitter users (n=3439) around the world were unsure about the safety of COVID-19 vaccines, in the polls conducted for 1 week in mid-February 2021. In addition, in the earlier analysis performed on manually coded tweets in Canada on December 18 and 23, 2020, a total of 48.3% (292/605) of tweets expressed concerns about the safety of vaccines [24,25]. These concerns are consistent with the public health focus as well as media attention to side effects including thrombosis and myocarditis (heart inflammation). This indicates the continued challenge for public health agencies to address concerns about vaccine safety and instill confidence about COVID-19 vaccines.

It is also interesting to note the lack of difference in sentiment toward the vaccine developed by pfizer versus that developed by AstraZeneca (Figure 3). This might suggest a more complex understanding of vaccination by Twitter users despite media attention toward people not wanting to be vaccinated with AstraZeneca’s vaccine in March 2021 [28,29]. The high attentiveness to dosing intervals after vaccination (Figure 3) further emphasizes the challenges of knowledge translation and science communication as evidence changes.

Our results (Figure 4) show that the discussion about vaccination gradually increased after vaccine rollout on December 14, 2020, and drastically decreased after mid-April 2021. This finding aligns with the deceleration in the uptake of the first vaccine dose [30,31]. This may indicate a shift of public attention away from vaccination and the need to bring back a focus on vaccination to enhance vaccine coverage.

The 500 most liked tweets showed more positive sentiments overall toward the key aspects outlined in Figure 6, especially toward vaccines and vaccination, compared to the sentiments from the remaining tweets. On closer inspection, the most liked or retweeted tweets (Table 3) showed an interesting dichotomy in Twitter users who reported a negative sentiment toward vaccines: the “anti-vaxxer” population that largely used negative sentiment as a means to discourage vaccination, and the “Covid Zero” population that also used negative sentiment, albeit to encourage vaccination, while also critiquing the public health response. This finding from the most influential tweets highlights the need for public health agencies to have a multipronged approach to health messaging related to vaccine information and misinformation. In addition, given that the most liked or retweeted tweets have a very large circle of influence (eg, 256,484 followers), investigating these tweets is crucial to identifying and tackling misinformation and even tracking dissemination of positive information. For example, if such tweets could be flagged for public health agencies through a dashboard, the information or claim in them could be verified or refuted in official channels.

### Limitations

This study highlights important trends in Twitter conversations, which may have crucial policy implications. However, the following limitations need to be considered when interpreting the results and when our pipeline is deployed in practice. First, the tweets we used might not be representative of the general population. Recently, it is reported that the largest age group in Twitter users is 30-49 years (44%) [32]. Second, it should be noted that specific cities might overrepresent Canada in our study according to previous work by Gore et al [33]. However, the Twitter data that meet our inclusion criteria are not sufficient for a city-level analysis, especially given that we carry out our analysis on the basis of aspects. The limitations we mention here exist not only for our study but also for most previous work, which used tweets for a certain period at a nationwide level.

Second, although the ABSApp approach for ABSA leverages manually edited domain-specific aspect and opinion terms to improve the accuracy of sentiment analysis, it is still premature to accurately capture the intricacy of human language, such as figurative languages. Nevertheless, these limitations are not particular to our study but rather inherent in all current state-of-the-art sentiment analysis tools and techniques as well.

### Conclusions

Social media presents a convenient opportunity to assess public perception on a wide variety of issues, as part of the pandemic response. This study examined public sentiments toward COVID-19 vaccination on tweets over an extended period in Canada. In addition, we also investigated the most influential tweets. Our findings could inform public health agencies to design and implement interventions to promote vaccination, and approach the goal of ending the pandemic.

## Abbreviations

ABSA: aspect-based sentiment analysis
